# Monte Carlo-based characterization of proton minibeam radiation therapy across clinically relevant beam parameters

**DOI:** 10.1016/j.phro.2026.101032

**Published:** 2026-07-04

**Authors:** Angela Corvino, Tim Schneider, Yolanda Prezado

**Affiliations:** a Institut Curie, Université PSL, CNRS UMR3347, Inserm U1021, Signalisation Radiobiologie et Cancer, 91400 Orsay, France; b Université Paris-Saclay, CNRS UMR3347, Inserm U1021, Signalisation Radiobiologie et Cancer, 91400 Orsay, France; c New Approaches in Radiotherapy Lab, Center for Research in Molecular Medicine and Chronic Diseases (CIMUS), Instituto de Investigación Sanitaria de Santiago de Compostela (IDIS), University of Santiago de Compostela, Santiago de Compostela, A Coruña, Spain; d Oportunius Program, Galician Agency of Innovation (GAIN), Xunta de Galicia, Santiago de Compostela, A Coruña, Spain

**Keywords:** Proton therapy, Dosimetry, Proton minibeam radiation therapy (pMBRT), Monte Carlo simulations

## Abstract

**Background and purpose:**

Proton minibeam radiotherapy (pMBRT) uses a 1D array of narrow beams to widen the therapeutic window of difficult-to-treat tumors. With the aim of identifying tumor locations that could benefit most from pMBRT, we evaluated how irradiation parameters shape 3D dose distributions.

**Materials and methods:**

Monte Carlo simulations were used to compute dose distributions in water for different proton energies, beam widths (bw*s*) and center-to-center distances (ctc*s*). Optimal parameter combinations were selected according to three criteria: (i) minimization of the bw in normal tissue; (ii) maximization of the valley dose in the target; and (iii) minimization of the peak dose in normal tissue.

**Results:**

For shallow tumors (≤ 2 cm), 0.5 mm beams with ctc = 3bw kept normal-tissue widths < 1 mm with Bragg-peak-to-entrance dose ratio (BEDR) > 1. For intermediate and deep-seated tumors (8–20 cm), 1.0–1.5 mm beams with ctc = 4–5bw kept normal-tissue widths < 7 mm with peak-to-valley dose ratio (PVDR) > 3 and achieved lateral dose homogeneity in the target. For very deep-seated tumors (> 20 cm), 2 mm beams with ctc = 4bw maintained normal-tissue widths < 10 mm with PVDR > 3 at the cost of BEDR ∼ 0.5.

**Conclusion:**

pMBRT may offer advantages over conventional proton therapy and GRID therapy for treating shallow and deep-seated tumors. For very deep-seated tumors (> 20 cm), feasibility will depend on tumor size and proximity of organs at risk.

## Highlights

• Monte Carlo map of proton minibeam radiotherapy across beam width, spacing and energy.
• Optimal parameter combinations reduce valley and peak doses in normal tissue.
• Optimal parameter combinations achieve target homogeneity.
• Potential advantages over conventional proton and proton grid therapy.

## Graphical abstract

**Figure:**
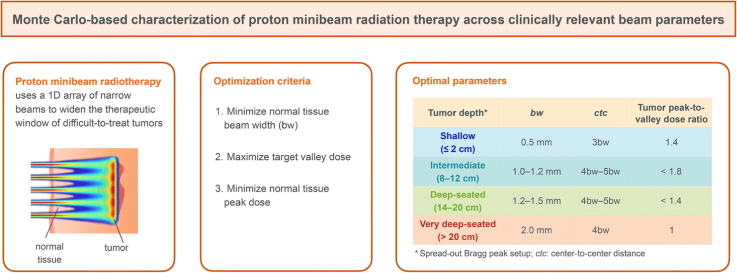

## 1 Introduction

Proton minibeam radiation therapy (pMBRT) is a spatially fractionated technique employing arrays of narrow proton beams, resulting in lateral dose profiles composed of high-dose areas (peaks) and low-dose areas (valleys) [1]. Very narrow beams allow the exploitation of dose-volume effects [2], [3]. Preclinical pMBRT studies in glioblastoma rat models have shown maintained or even enhanced tumor control [4], [5], [6], [7], together with a significant reduction in the neurological adverse effects associated with high radiation doses [7], [8], [9].

Clinically, pMBRT may represent an alternative dose-modulation strategy for high-grade gliomas and secondary brain metastases. The remarkably low normal-tissue adverse effects observed preclinically [8], [9], [10], [11] may allow more aggressive regimens while preserving cognitive function, particularly in pediatric oncology. Similar benefits could extend to radioresistant malignancies where local control is constrained by normal-tissue tolerance, including sarcomas, locally advanced bronchial cancer [12], and selected reirradiation cases. However, these potential advantages remain to be validated in clinical settings.

Treatment planning studies showed that pMBRT can provide target coverage comparable to stereotactic techniques while reducing integral dose to organs at risk (OARs) for brain tumors, liver and lung metastases [13], [14]. These results encourage the preparation of clinical trials [15]. While previous pMBRT dosimetric studies were typically limited to ∼ 100 MeV beams, 0.4–1.0 mm beam widths (bw*s*) and center-to-center distances (ctc*s*) up to 4.0 mm [16], [17], [18], [19], [20], [21], [22], [23], exploring higher energies (∼ 200 MeV) and wider beams (> 1 mm) is necessary to support clinical translation. Additionally, a comprehensive evaluation of how beam energy, bw, and ctc affect the 3D dose distribution, together with a biological interpretation of these parameters, is still lacking.

This study aimed to address this gap by expanding the parameter space explored in previous works and proposing dose-based criteria that may support improved biological outcomes. Monte Carlo simulations were performed to investigate the impact of beam energy, ctc, and initial bw on 3D dose distributions. Through this systematic investigation, we identified optimal irradiation geometries for the treatment of shallow, deep-seated, and very deep-seated tumors, and built a detailed dose-metric database to support pMBRT clinical implementation.

## 2 Materials and methods

### 2.1 Monte Carlo simulation details

The TOPAS toolkit [24] was used to compute dose distributions in water. No relative biological effectiveness (RBE) model was applied. The simulation parameters are summarized in Supplementary Material A (Table S1). Two single-beam geometries were investigated: an elongated rectangular mm-wide beam (MB geometry) and a square cm-wide beam (GRID geometry). One-dimensional arrays of rectangular beams (MB configuration) were also evaluated. Beam widths from 0.5 mm to 2.0 mm, proton energies from 50 MeV to 230 MeV, and ctc*s* from 3bw to 5bw were investigated. Simulations included pristine Bragg peak (PBP) setups and spread-out Bragg peak (SOBP) setups. SOBPs, with width set to one-quarter of the full range $R_{0}$, were generated by weighting seven monoenergetic beams [25]; the corresponding energies and weights are reported in Table S2.

### 2.2 Data analysis

The single beam 3D dose distributions (MB and GRID geometries) were analyzed to extract the lateral profile and the full width at half maximum (FWHM), used to characterize the beam broadening over depth. The 3D dose distribution relative to an array of rectangular beams (MB configuration) was also analyzed to obtain peak and valley depth–dose curves and the corresponding peak-to-valley dose ratio (PVDR). The Bragg-peak-to-entrance dose ratio (BEDR) was assessed for the peak depth–dose profiles. Peak and valley sampling positions are detailed in Fig. S1. To compare different MB configurations, we focused on dose homogeneity within the target. Based on the ICRU definition [26], [27], lateral homogeneity in the target region T was verified if:

(1) $$\forall \;\bar{z}\in T:\;0.95<\frac{\;D\left(\bar{z},y,x\right)}{D_{\mathrm{mean}}\left(\bar{z}\right)}<1.07,\forall x,y\in \mathrm{PTA}$$

where $D\left(z®,y,x\right)\;$is the local dose at position $\left(z®,y,x\right)$ and $D_{\mathrm{mean}}\;$is the mean dose in the planning target area (PTA = 2ctc×2ctc) at depth z®. $T=\left[R_{\mathrm{proximal}},R_{\mathrm{distal}}\right]$ for the PBP setup, and $T=\left[0.75R_{0},R_{0}\right]$ for the SOBP setup. A detailed description of the homogeneity analysis is provided in the Supplementary Material A.

### 2.3 Optimization criteria

Optimal parameter combinations for the MB configuration were identified using three criteria. First, the narrowest possible beam throughout the proton range was sought to maximize normal-tissue sparing via dose-volume effects and maintain a biological advantage over conventional radiotherapy (RT) and proton GRID therapy. Second, peak doses in normal tissue were minimized. When the beam is sufficiently narrow, as in microbeam radiation therapy (MRT), peak dose has minimal impact on normal tissue response, and the critical factors are the valley dose and the area covered by the valleys [28]. As the beam broadens in the minibeam radiation therapy (MBRT) range, the peak dose may play a significant role in sparing normal tissues, potentially becoming more relevant than valley dose [29], [30]. In MBRT, minimizing the normal-tissue peak dose corresponds to maximizing the BEDR. Third, the target valley dose was maximized, as it was identified as the dosimetric parameter most strongly correlated with increased survival in a retrospective evaluation of preclinical MRT and MBRT studies [31]. This criterion may translate into minimizing the PVDR in the target region.

## 3 Results

Optimal parameter ranges were identified for the MB configuration in PBP and SOBP setups (Table 1). In the complementary beam-broadening analysis (Supplementary Material B), rectangular sub-millimetric and millimetric beams (MB geometry) broadened by up to 20-fold, compared with < 2-fold for centimetric square beams (GRID geometry) over the same depth interval (Figs. S2 and S3). However, MB beams remained narrower than GRID beams across the full proton range for all investigated energies and setups (Fig. S4). Therefore, the subsequent analysis focused on the MB configuration, which enabled bw minimization and target dose homogeneity (Eq. (1)). For the PVDR ranges reported in Table 1, the lower bound corresponds to configurations satisfying Eq. (1), except at 50 MeV, where none of the evaluated parameter sets achieved target homogeneity. For energies ≥ 175 MeV, all combinations in Table 1 satisfied Eq. (1).

**Table 1:** Summary of the optimal parameter combinations relative to the MB configuration for both pristine Bragg peak (PBP) and spread-out Bragg peak (SOBP) setups across different initial energies. Abbreviations: MB, minibeam; bw, beam width; ctc, center-to-center distance; PVDR, peak-to-valley dose ratio; BEDR, Bragg-peak-to-entrance dose ratio.

**MB configuration - PBP setup**					
Energy [MeV]	bw [mm]	ctc	PVDR normal tissue ⁎	Mean PVDR target	BEDR

50	0.5	3bw	> 20	1.5	1.7
	0.7			3.5	2.4

125	1.0	4bw–5bw	> 15	1.0–1.1	1.1–0.9
	1.2			1.1–1.3	

175	1.2	5bw	> 4	1.1	1.0–0.8
	1.5	4bw–5bw		1.0–1.2	

230	2.0	4bw–5bw	> 4	1.0–1.1	0.8–0.7

**MB configuration - SOBP setup**					

Maximum Energy [MeV]	bw [mm]	ctc	PVDR normal tissue ⁎	Mean PVDR target	BEDR

50	0.5	3bw	> 30	1.4	1.3

125	1.0	4bw–5bw	> 4	1.1–1.4	0.6
	1.2		> 9	1.2–1.8	0.7–0.6

175	1.2	4bw–5bw	> 3	1.0–1.1	0.6–0.5
	1.5		> 4	1.0–1.4	

230	2.0	4bw	> 3	1.0	0.5

⁎ The PVDR in the normal tissue region was computed within the interval extending from the phantom entrance to half of the distal beam range.

**Fig. 1:**
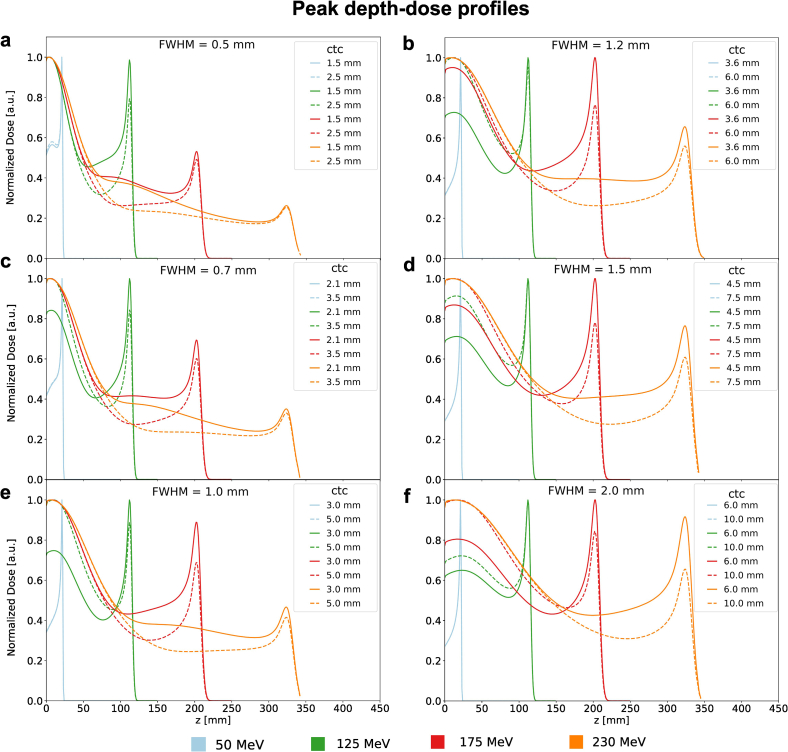
Peak depth-dose profiles for the MB configuration. The bw*s* (FWHM) are stated in the subplot titles. The dashed lines indicate the maximum ctc*s*, while the continuous lines represent the minimum ctc*s* considered in this study.

In the PBP setup, the BEDR was directly proportional to the bw and inversely proportional to the initial energy and the ctc (Fig. 1). For energies ≤ 50 MeV, the BEDR exceeded 1.7 across all simulated bw*s*. At higher energies, maintaining BEDR > 1 required proportionally wider beams.

**Fig. 2:**
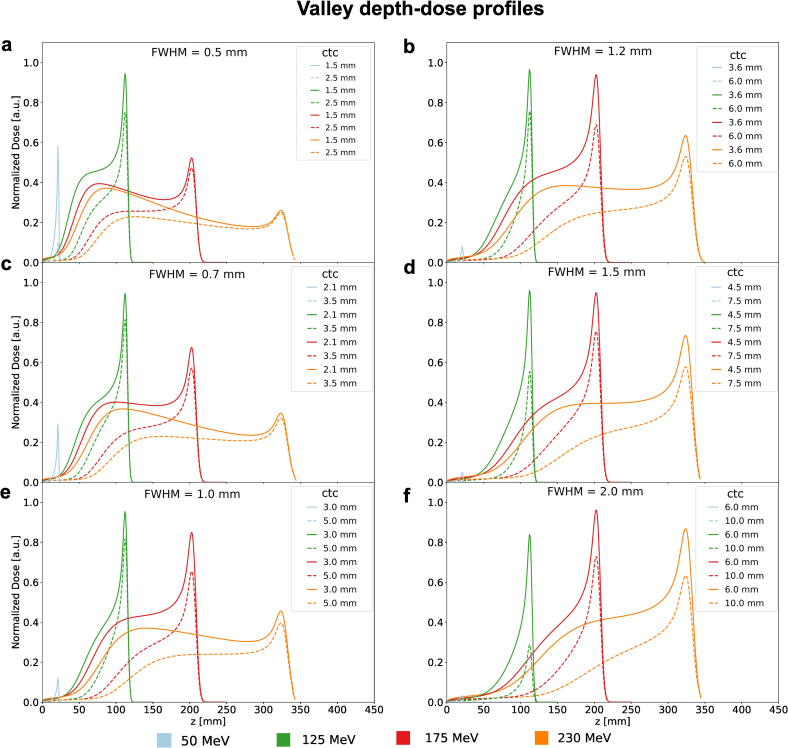
Valley depth-dose profiles for the MB configuration. The bw*s* (FWHM) are stated in the subplot titles. The dashed lines indicate the maximum ctcs, while the continuous lines represent the minimum ctcs considered in this study. The valley depth-dose profiles are normalized by the maximum of the corresponding peak depth-dose profiles.

For tumors located at depths ≤ 2 cm and energies ≤ 50 MeV, a trade-off between high BEDR and narrow beams was achieved with 0.5–0.7 mm widths. BEDR ranged from 1.7 to 2.4, independent of the ctc. A ctc/bw ratio of 3 maximized the target valley dose (Fig. 2(a)) while guaranteeing PVDR > 70 at the entrance and > 20 at shallow depths (Supplementary Material C, Figs. S5(a) and S5(c)). These combinations did not achieve target homogeneity with a single array (Supplementary Material D, Fig. S6).

For tumors located between 2 cm and 10 cm in water and beam energies of 50–125 MeV, the optimal bw ranged from 0.5 mm to 1.0 mm and increased with tumor depth and initial beam energy. A ctc < 4bw yielded BEDR > 1, whereas ctc ≥ 4bw steepened the proximal dose gradient and reduced valley doses upstream of the target and at intermediate depths (Fig. 2(a), (c), (e)). For 125 MeV, ctc = 3bw led to valley doses up to 45% of the BP dose at shallow–intermediate depths, whereas ctc = 5bw lowered them below 30% in the same region.

**Fig. 3:**
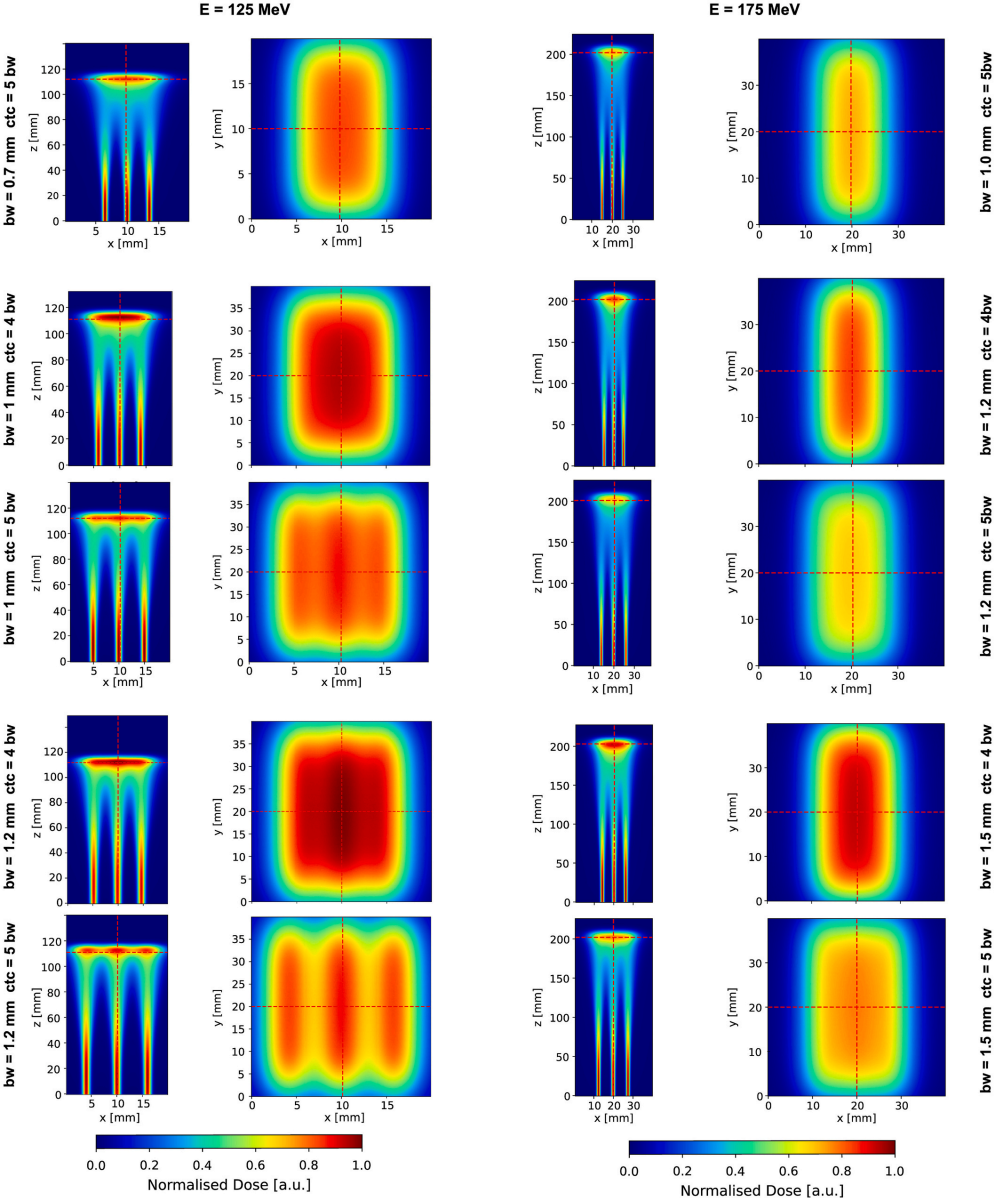
Dose distribution in water for an MB configuration with E = 125 MeV and E = 175 MeV. Left: longitudinal cross-section sampled at the central plane of the water phantom along the y-dimension (y = 1 cm and y = 2 cm). Right: lateral cross-section at the BP positions. The y-axis range is 2 cm for bw*s* < 1 mm and 4 cm for bw*s* ≥ 1 mm.

For tumors at 10 cm irradiated with 125 MeV, bw*s* of 0.7–1.2 mm offered a trade-off between beam-size minimization and BEDR maximization (1.0–1.35; Fig. 3, left panel). However, 0.7 mm beams yielded intermediate-depth valley doses exceeding 40% of the BP dose and were excluded from further consideration. For tumors at 20 cm irradiated with 175 MeV, bw*s* of 1.0–1.5 mm yielded favorable BEDR (0.8–1.1), but 1.0 mm beams produced the highest intermediate-depth valley doses and were thus excluded from further analysis. In both the 125 MeV and 175 MeV cases, ctc ≥ 4bw steepened the proximal dose gradient and reduced valley doses upstream of the target. Entrance PVDR remained > 100. Increasing the ctc from 4bw to 5bw preserved dose heterogeneity (PVDR > 1.2) 13–14% deeper along the beam path (Figs. S5(a) and S5(d)). At 125 MeV, target homogeneity was achieved with bw = 1.0–1.2 mm and ctc = 4bw (Figs. S5(b) and S5(e)); at 175 MeV, target homogeneity was achieved for all bw/ctc combinations shown in Fig. 3 (right panel).

**Fig. 4:**
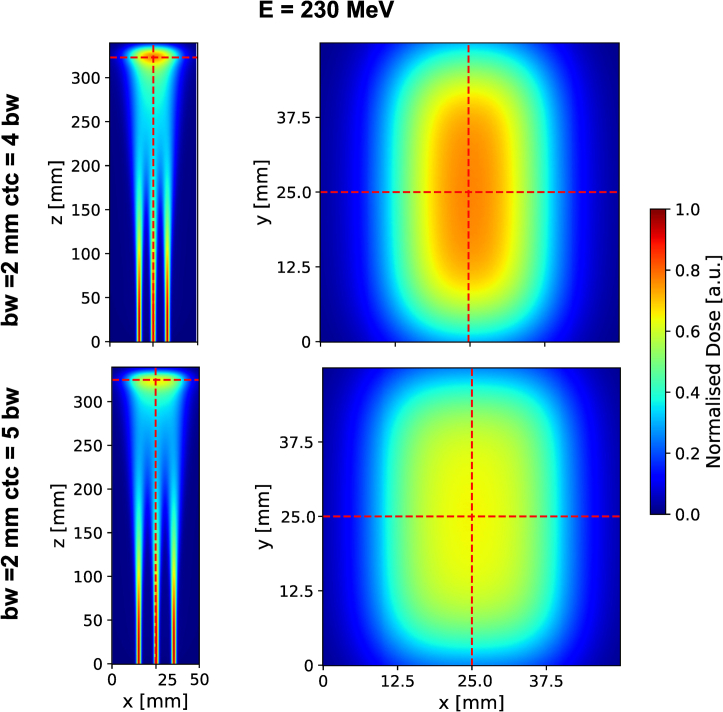
Dose distribution in water for an MB configuration with E = 230 MeV. Left: Longitudinal cross-section sampled at the central plane of the water phantom along the y-dimension (y = 2.5 cm). Right: Lateral cross-section at the BP position.

For tumors located at depths > 20 cm in water and energies of 175–230 MeV, bw > 1.2 mm and ctc ≥ 4bw were necessary to achieve BEDR ∼ 1 and maintain relatively low valley doses outside the target (Fig. 2(b), (d), (f)). At 230 MeV, bw = 2.0 mm achieved BEDR > 0.75. A ctc of 4bw minimized the entrance peak dose but led to earlier homogenization (∼ 20 cm), whereas ctc = 5bw kept PVDR > 1.2 until ∼ 25 cm (Fig. 4).

**Fig. 5:**
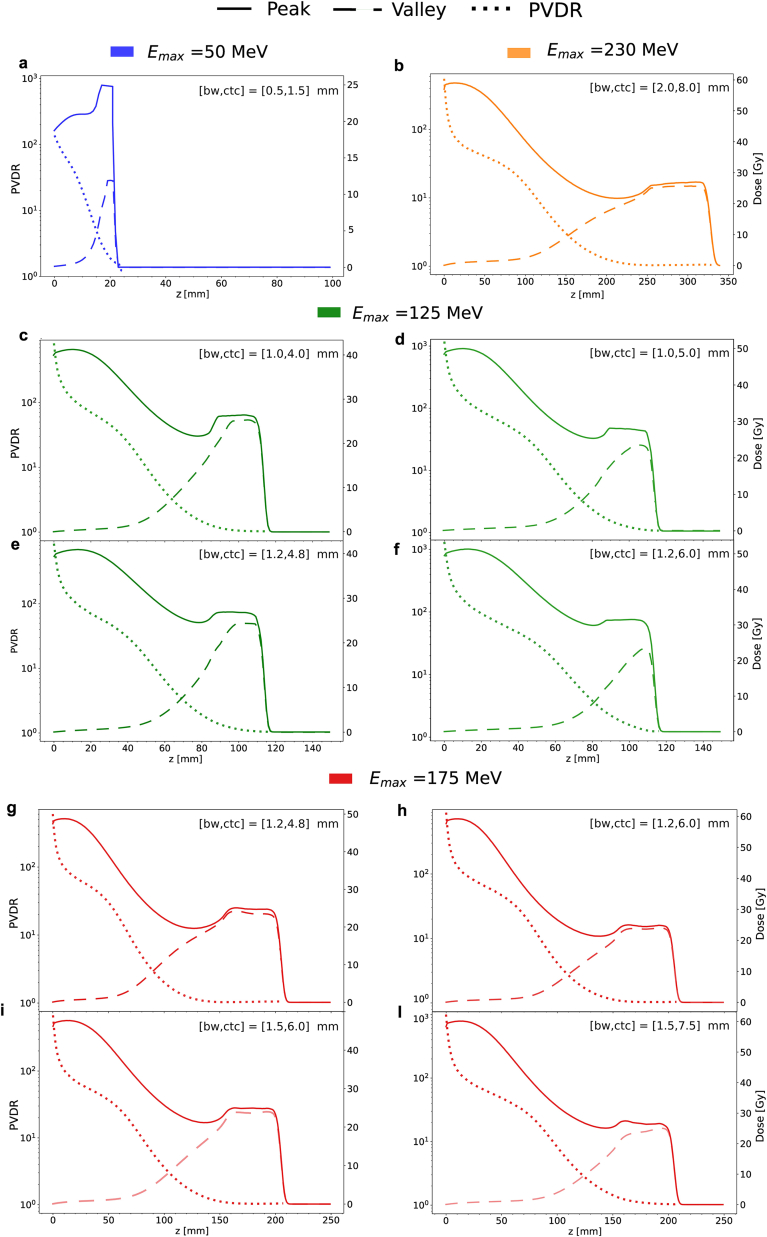
Depth–dose profiles for peak (continuous line) and valley (dashed line) region and PVDR (dotted line) as a function of depth for an SOBP setup and an MB configuration. All peak and valley profiles are normalized to deliver 25 Gy in the target region. Parameter combinations are: a) $E_{\max}$= 50 MeV, bw = 0.5 mm and ctc = 3bw; b) $E_{\max}$ = 230 MeV, bw = 2.0 mm and ctc = 4bw; c) $E_{\max}$ = 125 MeV, bw = 1.0 mm and ctc = 4bw; d) $E_{\max}$ = 125 MeV, bw = 1.0 mm and ctc = 5bw; e) $E_{\max}$ = 125 MeV, bw = 1.2 mm and ctc = 4bw; f) $E_{\max}$ = 125 MeV, bw = 1.2 mm and ctc = 5bw; g) $E_{\max}$= 175 MeV, bw = 1.2 mm and ctc = 4bw; h) $E_{\max}$= 175 MeV, bw = 1.2 mm and ctc = 5bw; i) $E_{\max}$= 175 MeV, bw = 1.5 mm and ctc = 4bw; l) $E_{\max}$= 175 MeV, bw = 1.5 mm and ctc = 5bw.

The optimal parameter combinations identified for monochromatic sources were reevaluated in SOBP setups. For shallow tumors (≤ 2 cm), a bw = 0.5 mm and ctc = 3bw were reevaluated for a 50 MeV maximum-energy SOBP (Fig. 5(a)). Compared with the monoenergetic case (Fig. 1(a)), BEDR decreased by 20%, while entrance PVDR increased by 60%. For tumors located at 8–12 cm, bw*s* of 1.0–1.2 mm with ctc = 4–5bw were reevaluated for a 125 MeV maximum energy SOBP (Fig. 5(c)–(f)). For a mean target dose of 25 Gy, peak doses at shallow and intermediate depths (z < 6 cm) ranged from 25 Gy to 50 Gy with bw < 2 mm. Although lower than in the monoenergetic case, BEDR remained > 0.6 for all configurations. A ctc of 4bw resulted in higher BEDR values and target dose homogenization (Fig. 5(c)–(f)). At 6 cm depth, bw = 1.0 mm yielded a valley dose of ∼ 9 Gy with PVDR of 4. In contrast, bw = 1.2 mm yielded a valley dose below 5 Gy and a PVDR = 6.

For tumors seated between 16 cm and 20 cm in water, bw*s* of 1.2–1.5 mm with ctc = 4–5bw were reevaluated for a 175 MeV maximum energy SOBP (Fig. 5(g)–(l)). While ctc = 4bw yielded the highest BEDR, all combinations achieved target homogeneity. A bw of 1.2 mm with ctc = 4bw balanced beam-size minimization and BEDR maximization, whereas bw = 1.5 mm reduced valley doses and increased PVDR at intermediate depths. At 10 cm depth, bw = 1.2 mm yielded a valley dose of ∼ 11 Gy and PVDR = 2, whereas bw = 1.5 mm reduced the valley dose to < 8 Gy and increased PVDR to 4. Valley doses within the first 4 cm remained < 1 Gy in all cases. For a mean target dose of 25 Gy, peak doses ranged from 40 Gy to 60 Gy within the first 5 cm with bw < 2 mm.

For tumors located at depths > 25 cm in water, MB configurations with bw = 2 mm were reevaluated. Under SOBP conditions (230 MeV maximum energy), ctc = 4bw yielded BEDR = 0.45 (Fig. 5(b)). For a mean target dose of 25 Gy, peak doses at shallow and intermediate depths (z < 10 cm) ranged from 40 Gy to 68 Gy, with PVDR values between 600 and 20 and valley doses within 0–2 Gy.

## 4 Discussion

In this Monte Carlo study, we analyzed how proton energy, bw, and ctc shape the 3D dose distribution in pMBRT for PBP and SOBP setups. pMBRT offered dosimetric and geometrical (bw) advantages over conventional proton therapy and proton GRID therapy for shallow, intermediate and deep-seated tumors (≤ 20 cm).

For shallow targets, such as subcutaneous metastases, ocular tumors [32], or superficial breast tumors [33], sub-millimetric beams preserved high spatial modulation in normal tissue but did not achieve target homogeneity with a single array. Homogeneity could be obtained by superimposing two arrays (e.g., a crossfire geometry [7]) or by interlacing them from two opposing/orthogonal directions [22], [34]. Two-dimensional pencil minibeam arrangements, including quadratic [17] and hexagonal [17], [35] patterns, have also been proposed. However, heterogeneous dose distributions may still achieve tumor control, as pMBRT studies in rat glioma reported significant tumor control with tumor PVDR as high as 6.1 [4], [5]. Immune activation and tumor microenvironment modulation may contribute to these effects [36], [37].

For tumors located between 2 cm and 10 cm in water, pMBRT optimal configurations involved 1.0–1.2 mm beams with potential applications in pediatric brain tumors, intermediate-depth gliomas [15], head and neck tumors, and breast tumors [15], [38]. Wider beams (1.2–1.5 mm) were more suitable for tumors located between 10 cm and 20 cm, including gliomas, meningiomas [13], pelvic sarcomas, large lung, liver or brain metastases [14]. For a mean prescribed target dose of 25 Gy, entrance and proximal peak doses ranged from 25 Gy to 60 Gy with bw < 2 mm. These values are comparable to those used in preclinical pMBRT [9] and X-ray MBRT [39] studies showing significant normal-tissue sparing. For a fixed bw, increasing beam energy reduced BEDR and target PVDR. A lower PVDR may favor tumor control through higher valley doses and improved target homogeneity but may also reduce normal-tissue sparing in proximal OARs. A lower BEDR (higher entrance peak doses) may attenuate the normal-tissue-sparing effect at the entrance. Wider beams and multifield approaches can mitigate this limitation if beam broadening remains limited enough to preserve dose-volume effects in normal tissues. Proton minibeam arc therapy could reduce peak and valley doses to normal tissues, as well as the volumes exposed to high-dose and high linear energy transfer (LET) values, compared with single-array pMBRT. However, treatment duration and delivery complexity should also be considered for such multifield approaches.

pMBRT with 2 mm beams could also represent a viable strategy for very deep-seated tumors (> 20 cm), including liver cancer [14], liposarcomas, retroperitoneal sarcomas [40], and abdominal metastases. Although these configurations delivered entrance peak doses higher than target peak doses (BEDR ∼ 0.5), they preserved spatial fractionation in normal tissues and achieved tumor dose homogeneity. High normal-tissue peak doses remained confined to very small volumes, with bw smaller than in proton GRID therapy. While normal tissue sparing with beams wider than 2 mm has not yet been experimentally validated, these configurations are expected to better exploit dose-volume effects than those employed in proton GRID therapy, potentially mitigating adverse effects from higher peak doses. Beyond dose-volume effects, rapid repair supported by preserved valley regions, migration of healthy cells from neighboring less-irradiated tissue, vascular and endothelial preservation [41], and reduced inflammation [11] may contribute to pMBRT normal tissue sparing, potentially enabling safer hypofractionated regimens with higher per-fraction doses [42]. For very deep-seated targets, however, integral dose and proximal normal-tissue adverse effects should be evaluated case by case, and multifield treatment planning and volumetric optimization may be needed to manage the high entrance dose associated with SOBPs. Large-animal or veterinary studies will also be needed to validate the potential advantages of pMBRT for these targets. Canine studies may provide a more realistic approximation to clinical conditions, as they involve spontaneous tumors, more comparable brain structures, megavoltage irradiation for deep-seated targets, and long-term follow-up. Treatment-planning studies in spontaneous canine brain tumors showed favorable dosimetric properties of X-ray MBRT and pMBRT [41], [43], and a randomized study reported better pathological tumor response and more limited normal-brain damage with MBRT than with conventional RT [41].

The interpretation of these results should consider several limitations. A single-field configuration was adopted to isolate the effects of proton energy, bw, and ctc on the 3D dose distribution, and target size was not evaluated as an independent variable. Therefore, field size effects were not explored. Larger targets, particularly those extending along the beam direction, would require broader SOBPs, which may alter the BEDR and, consequently, the optimal irradiation parameters. Larger irradiation areas and multi-field configurations may also lead to earlier overlap of minibeam arrays, potentially reducing normal tissue sparing. In addition, patient tissue heterogeneities and anatomical interfaces were not considered. Density and mass-thickness variations along the minibeam paths may affect proton range, lateral beam broadening, BEDR, and target homogeneity, potentially altering the optimal parameter combinations. Thus, the homogeneous water phantom used in this study should be interpreted as a baseline to identify general trends, to be refined in heterogeneous or patient-specific simulations and supported by experimental validation. Case-specific treatment-planning studies should also evaluate LET distributions and incorporate RBE models as a first conservative step toward biological optimization in pMBRT. The LQ model is expected to underestimate pMBRT tumor control efficacy and overestimate radiation-induced adverse effects.

In this study, proton minibeams were modeled as a magnetically focused source. Although magnetically focused proton minibeams may be feasible at clinical centers [20], current clinical implementations rely on mechanical collimators [44]. The collimator material may affect the 3D dose distribution and secondary neutron production, so radioprotection evaluations remain necessary for each beamline-specific pMBRT implementation. Tungsten can provide higher PVDR, whereas brass may offer a favorable compromise between PVDR, neutron contamination, and manufacturability [18]. At the Orsay Proton Therapy Center, the current multislit brass collimator [45] generates a clinically acceptable secondary neutron dose, higher than in conventional pencil beam scanning and lower than in passive-scattering proton therapy [46].

The results of this work could directly inform the design of upcoming phase I–II pMBRT clinical trials [1]. However, treatment planning, dose prescription, reporting, and dosimetric protocols will need to be adapted for clinical translation. Current clinical planning systems are optimized for uniform dose and cannot easily handle the unique objectives of spatially fractionated radiotherapy (SFRT), such as simultaneous peak and valley dose optimization. Custom Monte Carlo engines may provide interim solutions, but commercial platforms will eventually need to incorporate multi-objective optimization that accounts for spatial dose modulation. Regulatory and dosimetric standardization will also be required to define measurement protocols and acceptable dose metrics for SFRT. Early-phase clinical trials will be crucial to demonstrate safety and efficacy under carefully controlled conditions.

In conclusion, this in silico study indicates that pMBRT could enhance the therapeutic index of shallow and deep-seated tumors. For very deep-seated tumors, further experimental validation is required to establish feasibility and to assess potential advantages over proton GRID therapy, such as better exploitation of dose-volume effects.

## Data and code availability statement

The raw data supporting the conclusion of this article will be made available upon reasonable request to the corresponding author. The analysis scripts used for post-processing and figure generation are available in a public GitHub repository [47].

## CRediT authorship contribution statement

**Angela Corvino:** Writing – review & editing, Writing – original draft, Visualization, Software, Investigation, Formal analysis, Data curation. **Tim Schneider:** Writing – review & editing, Visualization, Supervision, Methodology. **Yolanda Prezado:** Writing – review & editing, Validation, Supervision, Resources, Project administration, Funding acquisition, Conceptualization.

## Declaration of competing interest

The authors declare that they have no known competing financial interests or personal relationships that could have appeared to influence the work reported in this paper.
