# Supplementary material for: Monte Carlo-based characterization of proton minibeam radiation therapy across clinically relevant beam parameters
Source: Phys Imaging Radiat Oncol. 2026 Jul 4;40:101032. doi: 10.1016/j.phro.2026.101032 (PMC13476367; doi:10.1016/j.phro.2026.101032)
Supplement: Supplementary file 1 [file mmc1.pdf]

Monte Carlo-based characterization of proton minibeam radiation therapy across clinically relevant beam parameters

Angela Corvino[1,2,⊥,*], Tim Schneider[1,+] and Yolanda Prezado[1,2,3,4]

The Supplementary Material is organized as follows. Supplementary Material A reports the Monte Carlo simulation details (Table S1), the generation of the SOBPs and the optimized energy weights for each clinically relevant configuration (Table S2), as well as the data analysis workflow, including the extraction of FWHM, peak and valley depth–dose profiles (Fig. S1), and the dose homogeneity analysis. Supplementary Material B presents the beam broadening evaluation for MB and GRID geometries in both PBP and SOBP setups (Figs. S2–S4). Supplementary Material C shows the evolution of PVDR with depth for representative MB configurations (Fig. S5). Supplementary Material D presents 3D dose distributions for specific combinations of the studied parameters (Fig. S6).

Supplementary Material A. Simulation and analysis methods

A.1 **Monte Carlo simulation details**

The simulations were performed using the TOPAS toolkit, and their main characteristics are summarized in Table S1. A magnetic collimation system was assumed to generate proton minibeams located at the entrance of a water phantom with variable lateral area (ranging from 2 × 2 cm^2 to 5 × 5 cm^2) and depth depending on the simulated beam energy and irradiation geometry. Two different beam geometries were investigated: an elongated rectangular mm-wide beam (referred to as MB geometry) and a square cm-wide beam (referred to as GRID geometry). The beam dimensions ranged from 500 µm × 2 cm to 2000 µm × 5 cm in the case of the MB geometry, and from 1 × 1 cm^2 to 2 × 2 cm^2 for the GRID geometry. The dose distribution resulting from a one-dimensional array of elongated rectangular sub-mm-wide and mm-wide beams was also investigated (referred to as the MB configuration). To save computation time, only a single beam was simulated, and the dose distributions were evaluated as the sum of the contributions of each individual minibeam. For each bw, several center-to-center distances (ctc*s*) were examined ranging from 3 × bw to 5 × bw with a step size of 0.1 mm.

Table S1. Summary of the main characteristics of the MC simulations, in accordance with the recommendation of AAPM TG-268 [1].

<table>
<tr><th>Item</th><th>Description</th></tr>
<tr><td>Codes</td><td>All simulations were performed using TOPAS version 3.6 released on the 1st of February 2021 and based on GEANT version 10.7.</td></tr>
<tr><td>Computational resources</td><td>All computing jobs were sent to the Joliot Curie-SKL supercomputer, which uses 2 × 24-core Intel Skylake@2.7GHz (AVX512) CPUs, with 2640 computing nodes in total, and 48 cores per node. The total time consumed for the simulations was 960 h. The simulation time refers to the full use of all 48 cores of a node.</td></tr>
<tr><td>Sources</td><td>Each source was simulated as an emittance source. A bigaussian shape was used with angular spread of 3 mrad.
<table>
<tr><th colspan="4">Source characteristics</th></tr>
<tr><td rowspan="2">$\sigma_x = \sigma_y$ [mm]</td><td>PBP setup</td><td colspan="2">SOBP setup</td></tr>
<tr><td>Energy [MeV/u]</td><td>Energy min [MeV/u]</td><td>Energy max [MeV/u]</td></tr>
<tr><td>0.213
0.297
0.425
0.510
0.637
0.850
4.25
6.37
8.50</td><td>50.0
125.0
175.0
230.0</td><td>42.5
106.25
148.75
195.5</td><td>50.0
125.0
175.0
230.0</td></tr>
</table></td></tr>
<tr><td>Number of primaries simulated</td><td>5.4×10^8</td></tr>
<tr><td>Cross sections</td><td>Standard Geant4 cross section data files were used from modules recommended modules for proton therapy [2–4]: g4em-standard_opt3, g4hphy_QGSP_BIC_HP, g4decay, g4ion-binarycascade, g4helastic_HP, and g4stopping. A range cut value of 10 µm was used for all particles in the water phantom.</td></tr>
<tr><td>Scored quantities</td><td>Doses were scored using the DoseToMedium discretized volume scorer. A maximum global uncertainty < 1% was obtained.
<table>
<tr><th>Dose accumulation voxels</th></tr>
<tr><td>0.05 mm × 0.05 mm × 1 mm for the MB geometry
0.5 mm × 0.5 mm × 1 mm for the GRID geometry</td></tr>
</table></td></tr>
<tr><td>Post-processing</td><td>Post-processing of the raw data was performed using Python (version 3.7) scripts to generate the relevant figures.</td></tr>
</table>

A.2 **SOBP generation**

SOBP was set to one-quarter of the full range. For this purpose, seven monoenergetic ion beams were weighted according to the method described by Jette and Chen [5], with the energies and relative weights detailed in Table S1. SOBP optimization was performed only for a subset of the investigated parameters, corresponding to clinically relevant parameter combinations. The weights were optimized so that:

$$\left|\frac{D(z)-D_{mean}}{D_{mean}}\right| < 0.05 \qquad \forall\, z \in T_{R_0} = [0.75\, R_0, R_0] \qquad \text{(S1)},$$

with R_0 being the range of the maximum energy and D_{mean} the mean dose inside the target region T_{R_0}.

Table S2 Energy and weighting factors for the composition of the SOBP.

bw = 0.5 mm ctc = 1.5 mm							
Energy [MeV]	42.5	43.82	45.11	46.37	47.6	48.81	50.00
Weighting factor	0.03	0.04	0.04	0.08	0.11	0.21	0.49
bw = 1.0 mm ctc = 4.0 mm							
Energy [MeV]	106.25	109.54	112.77	115.92	119.00	122.03	125.00
Weighting factor	0.03	0.03	0.06	0.07	0.12	0.18	0.51
bw = 1.0 mm ctc = 5.0 mm							
Energy [MeV]	106.25	109.54	112.77	115.92	119.00	122.03	125.00
Weighting factor	0.02	0.03	0.05	0.07	0.12	0.19	0.52
bw = 1.2 mm ctc = 4.8 mm							
Energy [MeV]	106.25	109.54	112.77	115.92	119.00	122.03	125.00
Weighting factor	0.02	0.03	0.05	0.08	0.11	0.19	0.52
bw = 1.2 mm ctc = 6.0 mm							
Energy [MeV]	106.25	109.54	112.77	115.92	119.00	122.03	125.00
Weighting factor	0.02	0.02	0.04	0.07	0.11	0.20	0.54

bw = 1.5 mm ctc = 6.0 mm							
Energy [MeV]	148.75	153.56	157.87	168.28	166.61	170.84	175.0
Weighting factor	0.02	0.05	0.06	0.07	0.1	0.18	0.52
bw = 1.5 mm ctc = 7.5 mm							
Energy [MeV]	148.75	153.56	157.87	168.28	166.61	170.84	175.0
Weighting factor	0.02	0.04	0.06	0.06	0.1	0.18	0.54
bw = 2.0 mm ctc = 8.0 mm							
Energy [MeV]	195.5	201.56	207.49	213.29	218.97	224.54	230.0
Weighting factor	0.03	0.03	0.04	0.07	0.09	0.18	0.56
bw = 2.0 mm ctc = 10.0 mm							
Energy [MeV]	195.5	201.56	207.49	213.29	218.97	224.54	230.0
Weighting factor	0.02	0.03	0.04	0.07	0.09	0.18	0.57

A.3 **Data analysis**

The simulated 3D dose distribution relative to a single beam was analysed for both MB and GRID geometries. To extract the lateral profile, the 3D dose was integrated over a region of interest (ROI) along one of the two lateral dimensions. The resulting 2D lateral dose profile was analysed at fixed depth and fitted with a Gaussian function, and the full width at half maximum (FWHM) was extracted. The FWHM was used to characterize the beam broadening over depth. The simulated 3D dose distribution relative to an array of rectangular beams (MB configuration) was also investigated. The 3D dose distribution was integrated over a square ROI in the lateral plane corresponding to the peak and valley regions, resulting in peak and valley depth-dose profiles. The sampling locations of the peak and valley regions are indicated in Fig. S1. Peak depth-dose profiles were normalized to their maximum. Valley depth–dose profiles were normalized to the maximum of the corresponding peak profiles. The BEDR was assessed for the peak depth–dose profiles. The PVDR was also assessed as a function of depth.

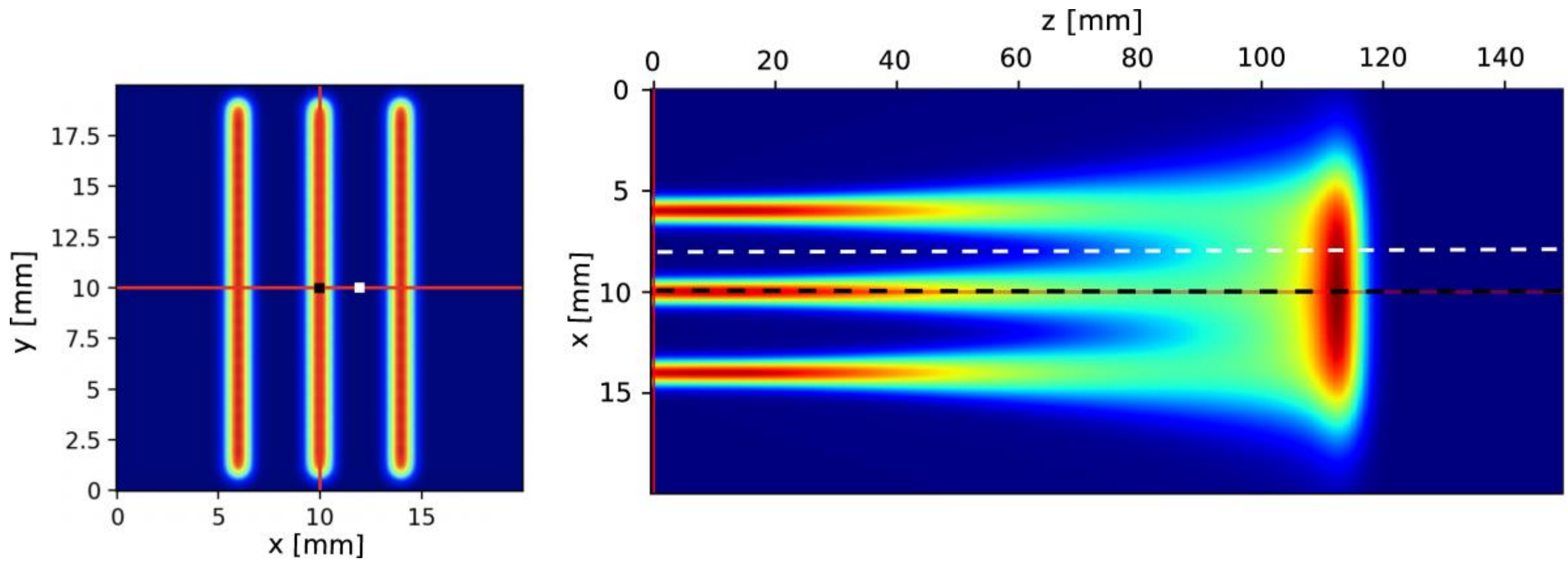

Fig. S1 Dose distribution in water for the MB configuration with E = 125 MeV. **Left**: lateral cross-section at the phantom entrance. **Right**: longitudinal cross-section at y = 1 cm. The black and white square/dashed lines indicate peak and valley regions, respectively.

We adopted the ICRU [6,7] definition of dose homogeneity to suit the unique characteristics of spatially fractionated radiotherapy. According to the ICRU, dose homogeneity is reached when the absorbed dose in the planning target volume (PTV) is confined within 95% to 107% of the prescribed dose. Since our focus is on the homogeneity of the 2D lateral dose distribution, we define the Prescribed Target Area (PTA) as $PTA = 2\,ctc \times 2\,ctc$. If we consider $D(z, y, x)$ as the 3D dose distribution scored in the water phantom, dose homogeneity in the lateral plane ($z = \bar{z}$) is verified if:

$$0.95 < \frac{D(\bar{z},y,x)}{D_{mean}(\bar{z})} < 1.07 \qquad \forall\, x, y \in \mathrm{PTA} \qquad \text{(S2)}.$$

In our study and for a PBP setup, we defined the target region as $T_R = [R_{proximal}, R_{distal}]$ where R is the depth of the proximal/distal point in the BP where the dose reaches 80% of the BP dose [8]. For monochromatic sources, if the Eq. (S2) is satisfied at z_0, it can be inferred that it will also be met at greater depths $z > z_0$, where increased scattering occurs. Therefore, it is sufficient to evaluate Eq. (S2) within a smaller interval $\mathrm{T}_{z_{BP}} = [R_{proximal}, z_{BP}]$ that corresponds to the proximal part of the Bragg peak. We considered homogeneity in the proximal part of the Bragg peak to be verified if:

$$\forall\, \bar{z} \in \mathrm{T}_{z_{BP}}: 0.95 < \frac{D(\bar{z},y,x)}{D_{mean}(\bar{z})} < 1.07, \quad \forall\, x, y \in \mathrm{PTA} \qquad \text{(S3)}.$$

In SOBP setups, the total dose is the sum of contributions from several monoenergetic beams:

$$D_{SOBP} = \sum_{i=0}^{n} D_{E_i}$$

where D_{E_i} is the dose contribution from a monoenergetic beam of energy E_i. Here, the target region is defined as $T_{R_0} = [0.75R_0, R_0]$ where R_0 is the (distal) range of the highest energy E_0. If the E_0 component is homogeneous at a given depth z_0, all lower-energy components are also homogeneous at z_0, since they undergo stronger multiple Coulomb scattering (lateral spread is inversely proportional to the speed of the incident particle). As a practical proxy, we evaluate homogeneity within the representative proximal SOBP interval $\mathrm{T}_{R_0} = [0.75R_0, 0.80R_0]$, and consider it verified if:

$$\forall\, \overline{z} \in \mathrm{T}_{R_0}: 0.95 < \frac{D(\bar{z},y,x)}{D_{mean}(\overline{z})} < 1.07, \quad \forall\, x,y \in \mathrm{PTA} \quad \text{(S4)}.$$

Supplementary Material B. Beam broadening evaluation

This section presents the beam broadening analysis for both MB and GRID geometries, in PBP and SOBP setups. Figs. S2 and S3 illustrate the evolution of the bw as a function of depth for the MB geometry (rectangular sub-mm and mm-wide beam) and the GRID geometry (square cm-wide beam), respectively. The bw is expressed in terms of FWHM. The beam broadening depends on beam energy and on tissue depth. Higher energetic beams broaden at a slower rate because particles have a greater forward momentum and are, therefore, less affected by lateral deflections due to multiple Coulomb scattering. Thus, at a fixed depth, the beam broadening percentage is smaller for higher-energy beams (see Figs. S2(b) and S3(b)). Although this effect is observable for all considered bws, it becomes less significant for cm-wide beams. For instance, at a depth of 10 cm in water, a 0.5 mm wide beam broadens to approximately 4 mm for an initial energy of 125 MeV, compared to 2 mm for a 230 MeV beam, indicating a 50% difference. In contrast, for a 2 cm wide beam, the difference is minimal: at a depth of 10 cm, a 230 MeV beam broadens to 2.1 cm, whereas a 125 MeV beam broadens to 2.3 cm, representing only a 9% difference.

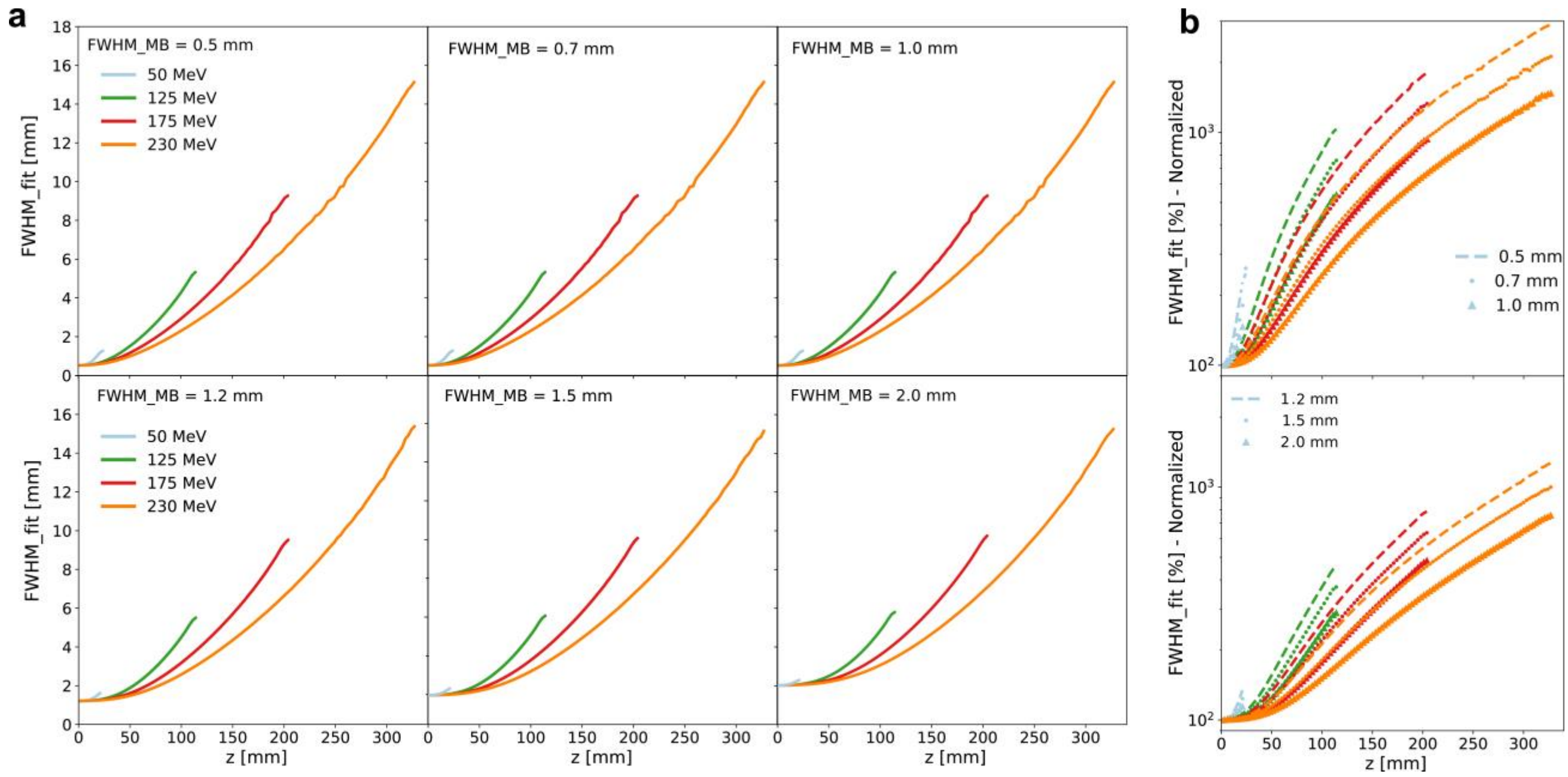

Fig. S2 Evolution of the FWHM as a function of depth for rectangular sub-millimetric and millimetric beams (**MB geometry**). The bw ranges from 0.5 mm to 1.0 mm in the first row and from 1.2 mm to 2.0 mm in the second row. **a)** Absolute FWHM values. **b)** Normalized FWHM values.

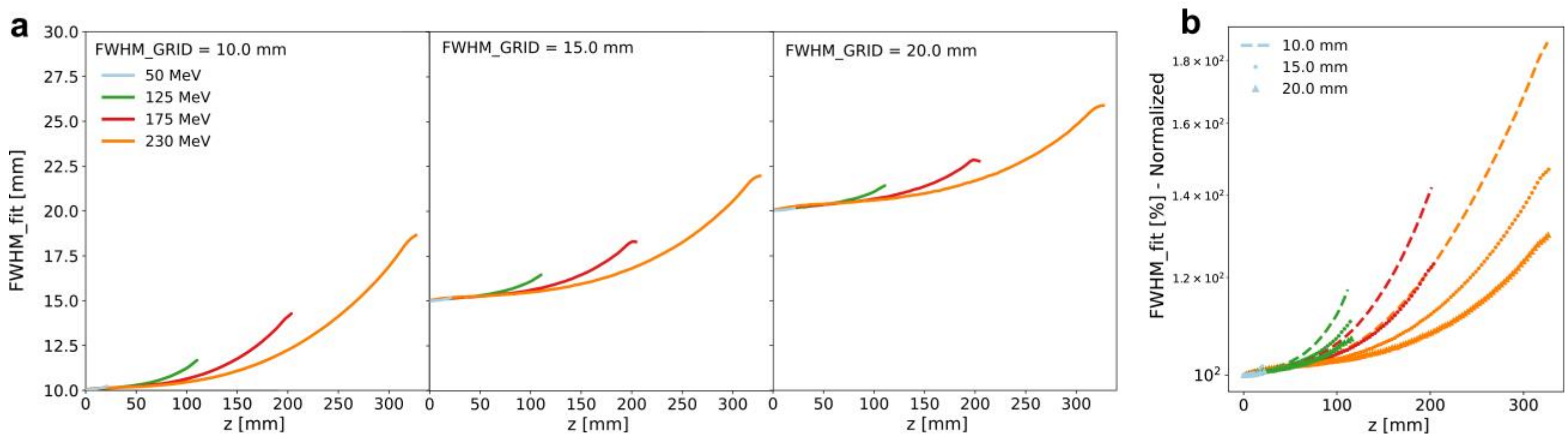

Fig. S3 Evolution of the FWHM as a function of depth for square cm-wide beams (**GRID geometry**). The bw varies from 1 cm to 2 cm. **a)** Absolute FWHM values. **b)** Normalized FWHM values.

Broadening is also inversely proportional to the initial bw, regardless of the beam shape, as visible in Figs. S2(b) and S3(b). Within the ranges explored in this study—0.5–2 mm for the MB geometry and 1–2 cm for the GRID geometry—broadening is markedly more pronounced for the MB geometry, with widths increasing by up to 20-fold over the beam range. In contrast, GRID beams widen by up to 1.8 times over the same range. For instance, at an initial energy of 125 MeV, a sub-millimetric rectangular beam broadens by a factor of 8–10, while a millimetric one widens by 3–5; a centimetric square beam under the same conditions only broadens by a factor of 1.2.

Despite the greater broadening in the MB geometry, bw*s* remain consistently narrower than those in the GRID geometry across the entire depth range and for nearly all energies considered in this study. For an initial energy of 50 MeV, a 0.5–2 mm wide rectangular beam broadens to approximately 1.5–2.2 mm FWHM at the end of range, remaining substantially narrower than the lateral dimensions characteristic of the GRID geometry. At 125 MeV, broadening reaches 5–6 mm FWHM, independently of the initial bw, and still remains below GRID values. Similarly, for 175 MeV, rectangular beams in the 0.5–2 mm range broaden to about 10 mm FWHM, while square beams in the GRID geometry with initial widths of 10–15 mm reach 14–18 mm FWHM under the same energy conditions. For 230 MeV, a 0.5–2 mm rectangular beam broadens to approximately 16 mm FWHM, while a 10–20 mm wide square beam broadens to 18–25 mm FWHM.

The significant beam broadening observed at the end of the range for high energies (E > 175 MeV) in the MB geometry might reduce the normal tissue benefits associated with the use of very narrow beams close to the target. However, the MB geometry consistently maintains narrow beam profiles at shallow depths (bw < 2 mm) and intermediate depths (bw < 6 mm), which favors normal tissue sparing through dose-volume effects. Additionally, the substantial broadening observed for submillimeter- and millimeter-scale beams can be effectively exploited in pMBRT to achieve higher valley doses and more homogeneous dose distribution within the target region compared to proton GRID therapy [9].

In addition to the PBP setup, SOBP configurations were also evaluated. The analysis focused on the MB geometry, as it proved more advantageous than the GRID geometry in the PBP context. For each clinically relevant proton energy, only the most advantageous beam widths, as identified in Section 3.2, were analyzed. Fig. S4 shows the evolution of the FWHM as a function of depth for a single rectangular minibeam and the corresponding subset of irradiation parameters.

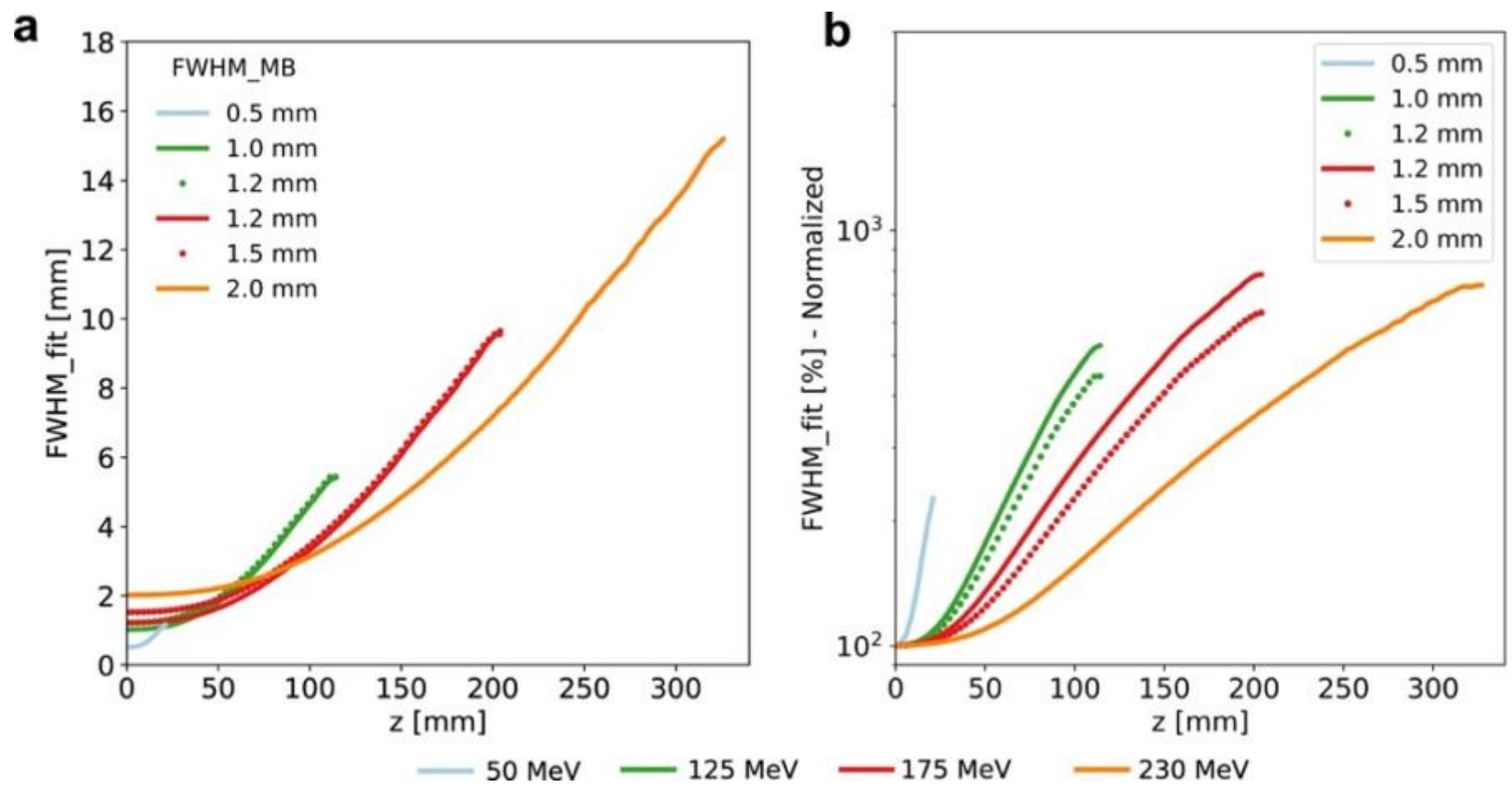

Fig. S4 Evolution of the FWHM as a function of depth for rectangular sub-millimetric and millimetric beams in the SOBP setup. The combinations of bw and energy correspond to the most clinically relevant cases identified in Table 1. **a)** Absolute FWHM values. **b)** Normalized FWHM values.

In the SOBP setups, at each depth, the beam consists of protons with different energies. Since lower-energy protons scatter more than higher-energy ones, greater beam broadening under SOBP conditions compared to PBP conditions could be expected. However, when comparing the lateral beam broadening between the PBP and SOBP setups while keeping the initial bw and maximum energy constant, no significant differences are observed. This suggests that the additional scattering induced by the mixed energy spectrum in the SOBP configuration has a negligible effect on the overall lateral beam spread. Thus, the considerations made for PBP setups remain valid under SOBP conditions. In particular, the beam widths used in MB geometry remain substantially smaller than those typical of GRID geometry throughout the entire beam range. As a result, MB configurations are expected to enhance normal tissue sparing via dose-volume effects, while simultaneously enabling a more homogeneous dose distribution within the target—an advantage not achievable with proton GRID therapy.

Supplementary Material C. PVDR evaluation

Fig. S5 illustrates the evolution of the PVDR with depth for an MB configuration and a subset of the investigated dosimetric parameters. The data correspond to the PBP setup. As expected, the PVDR decreases as a function of depth for all cases due to multiple Coulomb scattering. The PVDR evolution spans a two/three orders of magnitude interval across the beam range. This broad dynamic range

provides flexibility to achieve high PVDR values at shallow and intermediate depths and low values in the target, potentially enhancing normal tissue sparing while maintaining effective tumor coverage.

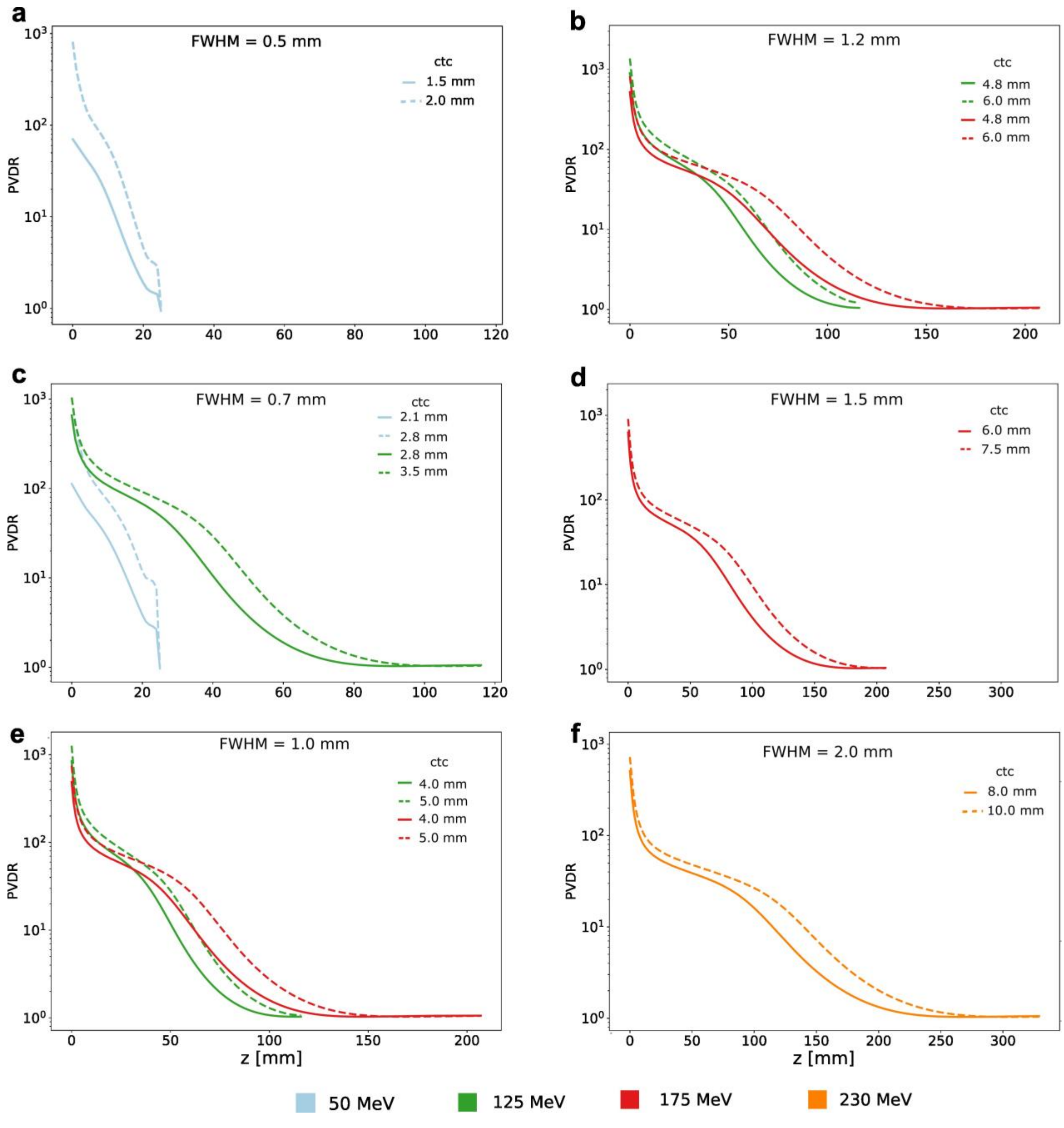

Fig. S5 PVDR as a function of depth and initial energy for the MB configuration. The bws (FWHM) are stated in the subplot titles. The dashed lines indicate the maximum ctcs, while the continuous lines represent the minimum ctc values of the optimal range identified in this study for each respective energy.

Supplementary Material D. 3D dose distributions

Fig. S6 shows the 3D dose distributions for the MB configurations identified as most advantageous for shallow tumors in the PBP setup (E = 50 MeV, bw = 0.5 and 0.7 mm, ctc = 3bw).

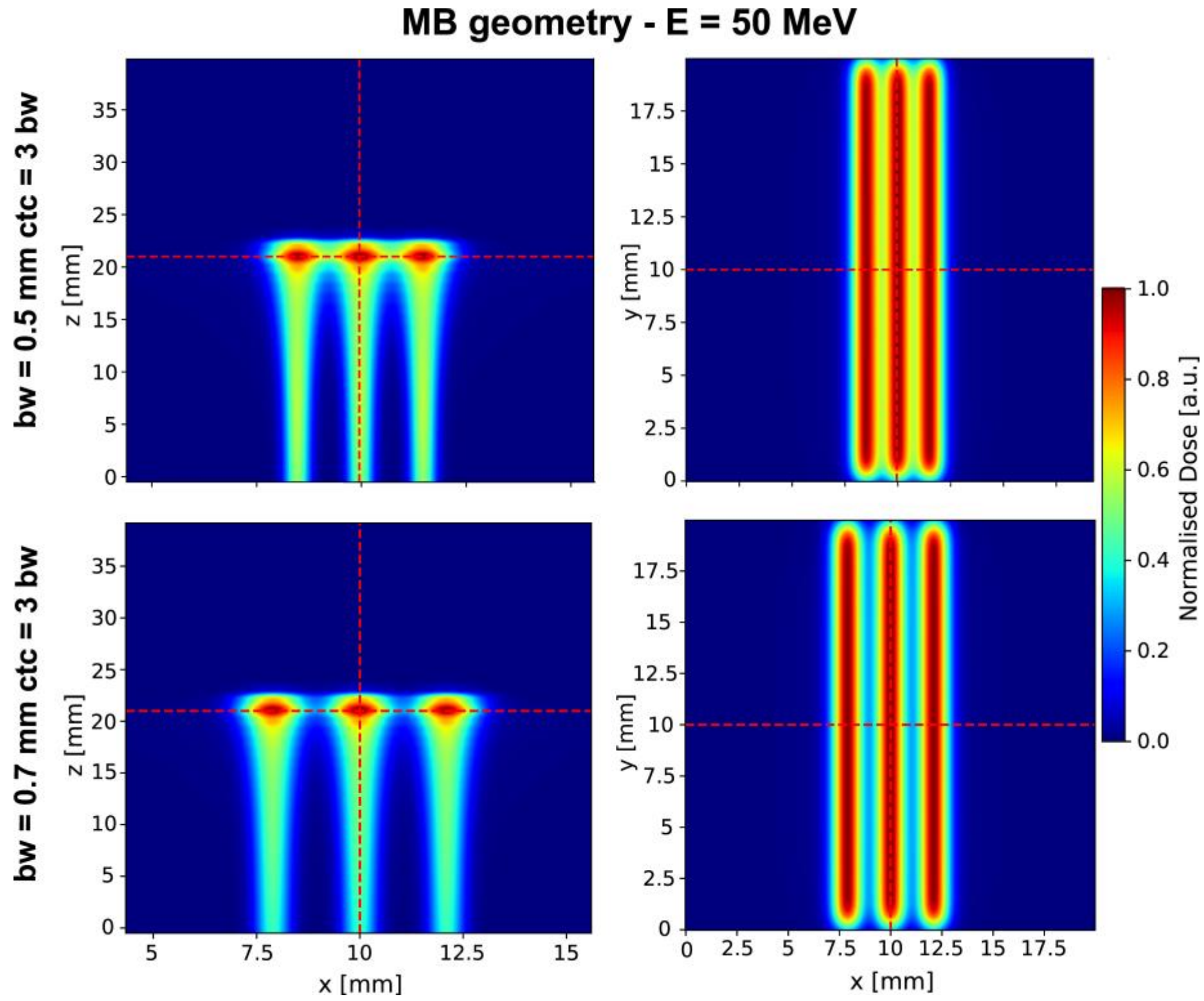

Fig. S6 Dose distribution in water for an MB configuration with E = 50 MeV, bw = 0.5,0.7 mm, and ctc = 3bw. **Left:** longitudinal cross-section sampled at the central plane of the water phantom along the y-dimension (y = 1 cm). **Right:** lateral cross-section at the BP position.

References

[1] Sechopoulos I, Rogers DWO, Bazalova-Carter M, Bolch WE, Heath EC, McNitt-Gray MF, et al. RECORDS: improved Reporting of montE CarlO RaDiation transport Studies: Report of the AAPM Research Committee Task Group 268. Med Phys 2018;45. https://doi.org/10.1002/mp.12702.

[2] Arce P, Lagares JI, Azcona JD, Aguilar-Redondo P-B. A proposal for a Geant4 physics list for radiotherapy optimized in physics performance and CPU time. Nucl Instrum Methods Phys Res Sect Accel Spectrometers Detect Assoc Equip 2020;964:163755. https://doi.org/10.1016/j.nima.2020.163755.

[3] Zacharatou Jarlskog C, Paganetti H. Physics Settings for Using the Geant4 Toolkit in Proton Therapy. IEEE Trans Nucl Sci 2008;55:1018–25. https://doi.org/10.1109/TNS.2008.922816.

[4] Testa M, Schümann J, Lu H -M., Shin J, Faddegon B, Perl J, et al. Experimental validation of the TOPAS Monte Carlo system for passive scattering proton therapy. Med Phys 2013;40:121719. https://doi.org/10.1118/1.4828781.

[5] Jette D, Chen W. Creating a spread-out Bragg peak in proton beams. Phys Med Biol 2011;56:N131–8. https://doi.org/10.1088/0031-9155/56/11/N01.

[6] The International Commission on Radiation Units and Measurements. ICRU-Report 83: Prescribing, Recording, and Reporting Photon-Beam Intensity-Modulated Radiation Therapy (IMRT). J ICRU 2010;10:NP.2-NP. https://doi.org/10.1093/jicru/ndq001.

[7] ICRU Report 62: Prescribing, Recording and Reporting Photon Beam Therapy (Supplement to ICRU Report 50). J ICRU 1999;32:1–52. Available from: https://www.icru.org/report/prescribing-recording-and-reporting-photon-beam-therapy-report-62.

[8] Bortfeld T. An analytical approximation of the Bragg curve for therapeutic proton beams. Med Phys 1997;24:2024–33. https://doi.org/10.1118/1.598116.

[9] Zhao X, Setianegara J, Hao Y, Mazur TR, Smith W, Liu J, et al. Dosimetry and Quality Assurance of a Phase I Trial of Proton GRID Therapy on a Compact Proton Therapy Delivery System. Int J Radiat Oncol Biol Phys 2024;120:e209. https://doi.org/10.1016/j.ijrobp.2024.07.472.
